# Human Infection with *Fusobacterium necrophorum* without Jugular Venous Thrombosis: A Varied Presentation of Lemierre's Syndrome

**DOI:** 10.1155/2017/5358095

**Published:** 2017-10-24

**Authors:** Muhammad Asim Rana, Yashwant Kumar, Abdullah Ali Lashari, Ahmed F. Mady

**Affiliations:** ^1^Critical Care Department, Bahria Town International Hospital, Lahore, Pakistan; ^2^Acute Medicine Department, King's Mill Hospital, Sutton-in-Ashfield, UK; ^3^Critical Care Department, King Saud Medical City, Riyadh, Saudi Arabia

## Abstract

Lemierre's syndrome is also known as postangina septicemia, which is commonly caused by *Fusobacterium necrophorum* also known as Necrobacillus and also by other microorganisms like *Staphylococcus*, *Streptococcus*, *Peptostreptococcus*, and *Bacteroides*. Though the disease starts as an upper respiratory tract infection, it may spread and cause thrombophlebitis of the internal jugular vein. It may present itself through cranial nerve palsy or sepsis involving distant organs like the lungs or bones. It is also known as forgotten disease because of its rarity. *Fusobacterium necrophorum* usually causes infection in animals and rarely affects humans. We hereby present a case of Necrobacillus infection which did not cause any thrombophlebitis but resulted in severe pneumonia and acute kidney injury, leading to respiratory failure and requiring mechanical ventilation.

## 1. Case Report

**Figure 1:**
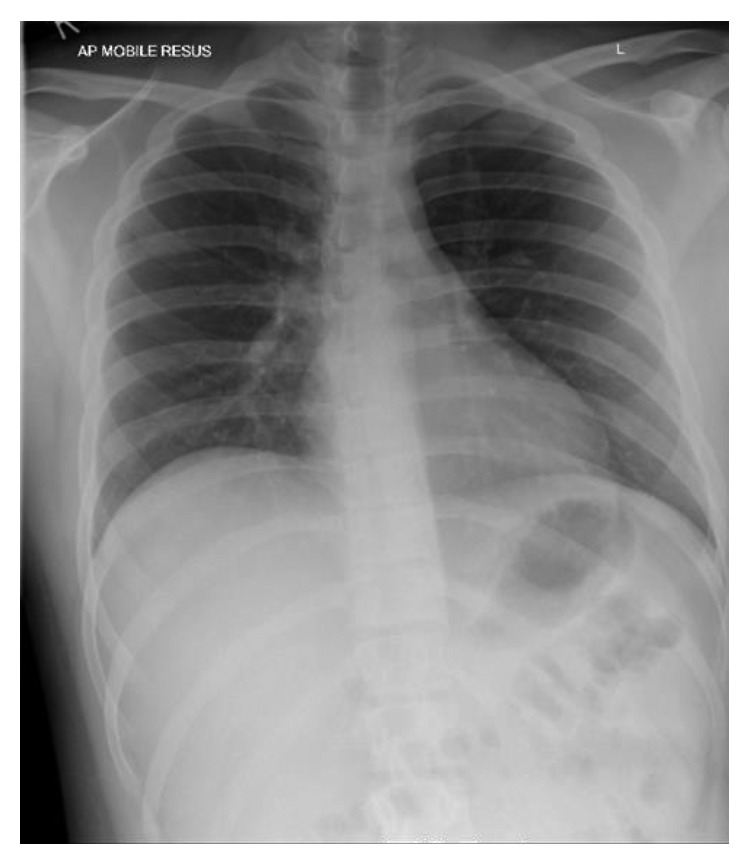
First chest X-ray (normal).

A 16-year-old male presented to the emergency room with a two-week history of sore throat, shortness of breath, and spiking fever. His physical findings were unremarkable, except for the temperature of 103°F and his SpO_2_ of 89% on room air. His initial white cell count, LFTs, and coagulation profile were within normal limits as was his chest X-ray (Figure 1()). The only findings were mild anemia, mildly raised bilirubin, and raised LDH. Striking lab results were creatinine raised to a value of 277 μmol/L, urea 94 mg/dL, and D-dimers 13,970 µg/L. He was admitted considering the differentials of sepsis-induced acute kidney injury, TTP, and pulmonary-renal syndrome, though his normal white cell count and normal chest X-ray were confusing.

**Figure 2:**
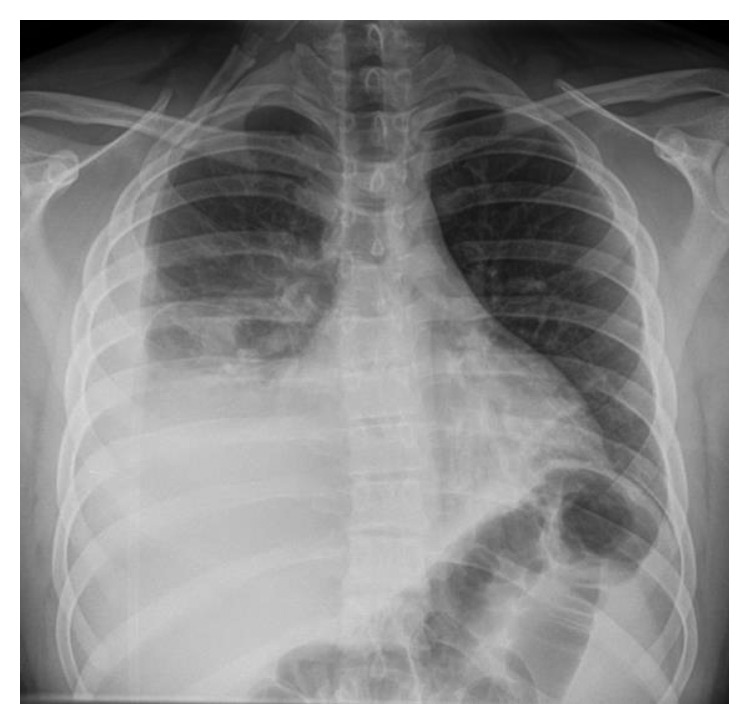
Second chest X-ray (after 48 hours). Right-sided severe pneumonia with parapneumonic effusion.

**Figure 3:**
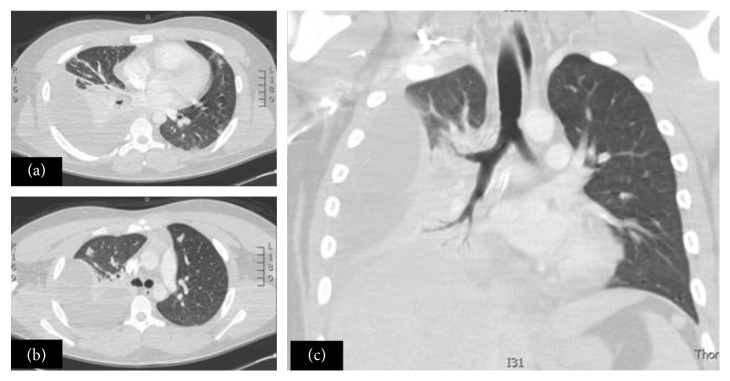
(a) and (b) CT scan chest (severe consolidation with effusion). (c) Coronal image of CT chest showing extensive consolidation and para pneumonic effusion.

In the next 48 hours, his condition worsened with dyspnea and hypoxia. His saturation dropped to 70% despite supplemental oxygen. White cell counts (WCCs) jumped to 24 × 10^9^/L. An urgent chest X-ray was carried out which was horrendous (Figure 2()), showing clear extensive consolidation. The patient was transferred to the intensive care unit for the sake of mechanical ventilation to combat hypoxia. The contrast-enhanced computerized tomographic scan of his chest showed extensive consolidation on his right side with parapneumonic effusion (Figures 3()–3()).

An extensive workup in search of diagnosis was carried out, including ANA, anti-GBM antibodies, infectious mononucleosis, pneumococcal antigen, *Legionella pneumophila* antigen, influenza A and B, and respiratory syncytial virus, but all these were negative. Though his pleural fluid was exudative, the Gram and ZN stains were negative as well.

After incubation for 21 hours, his anaerobic blood culture showed growth of *Fusobacterium necrophorum* sensitive to metronidazole for which appropriate antibiotic switch was done.

**Figure 4:**
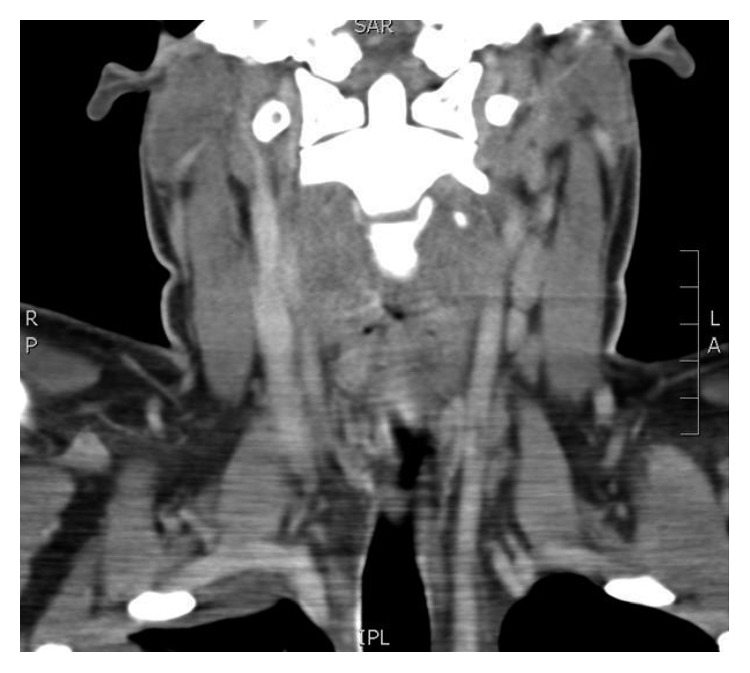
CT neck. No venous thrombosis.

Due to an infection with *Fusobacterium necrophorum*, Lemierre's syndrome (forgotten disease) was considered, and a search for jugular venous thrombosis was carried out and a CECT neck was done which did not show any thrombosis (Figure 4()).

The patient stayed in the intensive care unit for ten days but eventually showed remarkable improvement and was successfully weaned off and extubated.

Human infection with *Fusobacterium necrophorum* once called forgotten disease is on the rise again. This case highlights this fact and elaborates a varied presentation of severe pneumonia due to Necrobacillus but without jugular venous thrombosis.

## 2. Discussion

Lemierre's syndrome or postanginal septicemia was previously called “forgotten disease” because it was extremely rare [1]. Incidence of Lemierre's syndrome has increased over the last decade because of increasing antibiotic resistance, reducing rates of tonsillectomy, and better diagnostic tools [2–4]. Lemierre's syndrome is most commonly caused by *Fusobacterium necrophorum*, but other organisms, for example, *Staphylococcus*, *Streptococcus*, *Proteus*, *Bacteroides*, and *Peptostreptococcus*, can also cause Lemierre's syndrome [5].

This syndrome most commonly occurs in young adult males (our patient is a typical example) but can affect any age group. The disease begins in the tonsils and peritonsillar area and manifests as oropharyngeal symptoms with cervical lymph node enlargement [6]. The disease may then spread and cause thrombophlebitis of the internal jugular vein and cranial nerve palsy [6].

In late stages, the infection can spread through the bloodstream and involve the lungs or bones [5, 6]. Sepsis usually sets in after oropharyngeal symptoms have resolved and hence the term “postanginal sepsis” [7]. Septic emboli can occur most commonly in the lungs causing lung abscess or empyema but may also be found in the muscles, bones, brain, and liver [5, 8].

Risk factors for development of internal jugular vein thrombosis are central line catheterization, hypercoagulability, and infection [9].

Considering diagnostic criteria, it is largely based on suspicion depending upon the patient's history, signs, and symptoms. If a persistent sore throat with symptoms is found, physicians are cautioned to screen for Lemierre's syndrome. Laboratory investigations reveal signs of bacterial infection with raised WCC (neutrophilia), raised C-reactive protein (CRP), and raised erythrocyte sedimentation rate (ESR). Platelets may go either way, low or high. The liver and renal function tests are often abnormal. Gold standard is blood culture showing *Fusobacterium necrophorum* which may take 6–8 days [6]. Other tools which can be used to diagnose the condition are ultrasonography, CT scan, MR angiography, gallium scan, and radionuclide venography with Tc-99m-labelled RBC. USG helps in identifying internal jugular vein thrombus while CT scan shows distended neck veins with swelling of adjacent soft tissues. CT scan also helps in identifying metastatic lesion as in the lungs [8, 10, 11].

Early diagnosis and use of appropriate intravenous antibiotics have a good outcome. Penicillin group of antibiotics and antibiotics with good anaerobic coverage, for example, metronidazole and clindamycin, are effective in Lemierre's syndrome [7, 12]. Duration of antibiotics can vary from 2 weeks to 3 months depending on the extent of the disease and clinical response [13, 14].

Role of anticoagulation in patients with Lemierre's syndrome is controversial [7, 15]. Surgical modalities like venous ligation and resection of thrombosed veins are indicated if patients fail to respond to antibiotics. In general, prognosis for full recovery is good if prompt and appropriate treatment is given. A mortality rate of 4–18% has been recorded in the past [6, 16].

This case highlights the rise of human infection with *Fusobacterium necrophorum* which had almost disappeared from the world and was labelled as forgotten disease. It is important to emphasize that even without internal jugular thrombosis, distant (from throat) septic complications can occur as has been reported in our case. The term “incomplete Lemierre syndrome” has been proposed in such cases by Shiber et al. [17].
